# Adjustments of γδ T Cells in the Lung of *Schistosoma japonicum*-Infected C56BL/6 Mice

**DOI:** 10.3389/fimmu.2020.01045

**Published:** 2020-06-04

**Authors:** Hefei Cha, Hongyan Xie, Chenxi Jin, Yuanfa Feng, Shihao Xie, Anqi Xie, Quan Yang, Yanwei Qi, Huaina Qiu, Qiongli Wu, Zhinan Yin, Jianbing Mu, Jun Huang

**Affiliations:** ^1^Guangdong Provincial Key Laboratory of Allergy and Clinical Immunology, Sino-French Hoffmann Institute, The Second Affiliated Hospital of Guangzhou Medical University, Guangzhou, China; ^2^Department of Immunology, Zhongshan School of Medicine, Sun Yat-sen University, Guangzhou, China; ^3^Biomedical Translational Research Institute, School of Pharmacy, Jinan University, Guangzhou, China; ^4^Laboratory of Malaria and Vector Research, National Institute of Allergy and Infectious Diseases, National Institutes of Health, Bethesda, MD, United States

**Keywords:** *Schistosoma japonicum*, lung, γδ T cells, phenotype, function

## Abstract

Many kinds of lymphocytes are involved in *Schistosoma japonicum* (*S. japonicum*) infection-induced disease. γδ T cells comprise a small number of innate lymphocytes that quickly respond to foreign materials. In this study, the role of γδ T cells in the lung of *S. japonicum*-infected C56BL/6 mice was investigated. The results demonstrated that *S. japonicum* infection induces γδ T cell accumulation in the lung, expressing higher levels of CD25, MHCII, CD80, and PDL1, and lower levels of CD127 and CD62L (*P* < 0.05). The intracellular cytokines staining results illustrated higher percentages of IL-4-, IL-10-, IL-21-, and IL-6-producing γδ T cells and lower percentages of IFN-γ-expressing γδ T cells in the lung of infected mice (*P* < 0.05). Moreover, the granuloma size in lung tissue was significantly increased in Vδ^−/−^ mice (*P* < 0.05). In the lung of *S. japonicum*-infected Vδ^−/−^ mice, both type 1 and type 2 immune responses were decreased significantly (*P* < 0.05). In addition, the expression of CD80 and CD69 on B cells was decreased significantly (*P* < 0.05), and the SEA-specific antibody was markedly decreased (*P* < 0.05) in the blood of infected Vδ^−/−^ mice. In conclusion, this study indicates that γδ T cells could adjust the Th2 dominant immune response in the lung of *S. japonicum*-infected mice.

## Introduction

Schistosomiasis, caused by blood flukes of the genus *Schistosoma*, is the second most common endemic parasitic disease in the world (1). More than 260 million people live with schistosomiasis, and regular mass treatment is used to prevent the disease (2). *Schistosoma japonicum* (*S. japonicum*), which infects both humans and mice, is commonly used to study *Schistosoma* infection in murine models. After *S. japonicum* infection, the parasite transitions through a number of tissues, including the lung. Once reaching the adult stage, the fluke lays eggs, which are deposited in the liver, lung, and intestinal wall, inducing granulomatous inflammation, and progressive fibrosis (3, 4).

The lung is an important respiratory organ in human and other animals, and a plethora of immune cells reside in the lung, including T helper (Th) cells, natural killer (NK) cells, natural killer T (NKT) cells, gamma delta T cells (γδ T cells), myeloid-derived suppressor cells (MDSCs), macrophages, and others (5, 6). Interestingly, the lung is reportedly a niche for hematopoietic progenitors, which produce platelets and other immune cells (7, 8).

γδ T cells comprise a small number of innate lymphocytes that quickly respond to foreign materials without the need for antigen presentation (9). γδ T cells mediate the production of inflammatory cytokines, including interferon-γ (IFN-γ), tumor necrosis factor-α (TNF-α), and interleukin (IL)-17, thus participating in whole body or local immune regulation (10). γδ T cells also express high levels of cytotoxic molecules, such as granzyme A, granzyme B, and Fas-ligand (11). In the early stages of the immune response, γδ T cells are the main source of IL-17 and play a key role in the body's defense against bacterial invasion (12). IL-17 has potent pro-inflammatory functions, including the induction of IL-6 and TNF-α, as well as the recruitment and enhancement of neutrophils (13).

Dendritic cells (DCs), monocytes/macrophages, and B cells are professional antigen presenting cells (APCs), which process and present foreign antigens, activate classic T and B cells, and modulate the type of immune response. Recent reports demonstrated that activated γδ T cells could increase the expression of CD80, CD86, and HLA-DR (14), acting as the antigen-presenting cells that initiate the immune response, essentially bridging innate and adaptive immunity (15). Skin, adipose tissues, and mucosal tissues such as lung and intestine are sites where these cells are enriched (16). It has been reported that γδ T cells play an essential role in the defense against external pathogens, including viruses, bacteria, and parasites (17). γδ T cells appear to be a first line of defense against pathogen invasion (18) and may be involved in the establishment and regulation of the inflammatory response (19). In mice infected with *Staphylococcus aureus*, γδ T cells in the lung are not only beneficial for the removal of bacteria, but also contribute to the repair of damaged tissue (20). Importantly, as a potent effector cells, γδ T cells are used for the treatment of cancer, such as non-small cell lung cancer and leukemia (21, 22). Therefore, elucidation of biological characteristics of γδ T cells will be helpful for the further clinical application of γδ T cells. In our previous study, γδ T cell was found involved in the immune response in the liver and mesenteric lymph nodes of *S. japonicum* infected mice (10), but the lung was not studied. Thus, the purpose of this study was to identify the potential roles of γδ T cells during *S. japonicum* infection in C57BL/6 mouse lungs.

## Materials and Methods

### Mice

Six- to eight-weeks old female C57BL/6 mice were purchased from Traditional Chinese Medicine University of Guangzhou Animal Center (Guangzhou, China), and Vδ^−/−^ mice (B6.129P2-Tcrd^tm1Mom^/J, C57BL/6J genetic background) were obtained from JAX Stock (No. 002120). All animal experiments were performed in strict accordance with the Regulations for the Administration of Affairs Concerning Experimental Animals (1988.11.1). All protocols for animal use were approved to be appropriate and humane by the institutional animal care and use committee of Guangzhou Medical University (2012-11). Every effort was made to minimize suffering.

### Infection

C57BL/6 and Vδ^−/−^ mice were percutaneously infected with 40 ± 5 *S. japonicum cercariae* obtained from infected *Oncomelania hupensis* snails (purchased from Chinese Institute of Parasitic Disease, Shanghai, China) and euthanized 5 or 6 weeks after infection. Pathogen-free C57BL/6 and Vδ^−/−^ mice were used as controls.

### SEA and SWA

*S. japonicum cercariae*, SEA, and SWA were obtained from Jiangsu Institute of Parasitic Diseases (China). SEA and SWA were sterile-filtered, and the endotoxin was removed using Polymyxin B agarose beads (Sigma, USA). A limulus amebocyte lysate assay kit (Lonza, Switzerland) was used to confirm the removal of the endotoxin from SEA and SWA, as previously described (23).

### Lymphocyte Isolation

Mice were euthanized at week 5 or 6 post-infection. Before obtaining the lung tissue, blood was collected, mice were perfused with sterile saline to remove blood from the body. Excised lung tissue was cut into small pieces and incubated in 5 ml of digestion buffer (collagenase IV/DNase I mix, Invitrogen Corporation) for 30 min at 37°C and 5% carbon dioxide. Digested lung tissue was pressed through a 200-gauge stainless-steel mesh and was then suspended in Hank's balanced salt solution (HBSS). Lymphocytes were isolated using Mouse Lymphocyte Separation Medium (DAKEWE, China) density gradient centrifugation. Isolated cells were washed twice in HBSS and re-suspended at 1.5 × 10^6^ cells/ml in complete RPMI 1640 medium supplemented with 10% heat-inactivated fetal bovine serum (FBS), 100 U/ml penicillin, 100 μg/ml streptomycin, 2 mM glutamine, and 50 μM 2-mercaptoethanol. Single lung cell suspensions were prepared for flow cytometry analysis.

### Antibodies

APC-cy7-conjugated anti-mouse CD3 (145-2C11), FITC-conjugated anti-mouse γδ TCR (GL3), PE-conjugated anti-mouse CD8 (53-6.7), PerCP-cy5.5-conjugated anti-mouse CD4 (RM4-5), PE-conjugated anti-mouse CD25 (3C7), Brilliant Violet 421-conjugated anti-mouse CD274 (MIH5), APC-conjugated anti-mouse CD273 (TY25), PE-conjugated anti-mouse CD183 (CXCR3-173), APC-conjugated anti-mouse CD184 (2B11/CXCR4), PerCP-conjugated anti-mouse CXCR6 (FAB2145C), PE-conjugated anti-mouse IL-4 (11B11), PE-conjugated anti-mouse IL-17 (TC11-18H10), PE-conjugated anti-mouse IL-10 (JES5-16E3), APC-conjugated anti-mouse IL-5 (TRFK5), PE-conjugated anti-mouse IL-2 (554428), and isotype-matched control monoclonal antibodies (X39, G155-178) were purchased from BD Pharmingen (San Diego, CA, USA). PE-conjugated anti-mouse CD3 (145-2C11), Brilliant Violet 510-conjugated anti-mouse γδ TCR (GL3), APC-conjugated anti-mouse CD4 (RM4-5), PE-cy5-conjugated anti-mouse CD19 (6D5), PE-conjugated anti-mouse Vγ2 (A7R34), APC-conjugated anti-mouse CD69 (H1.2F3), PE-conjugated anti-mouse CD127 (A7R34), APC-conjugated anti-mouse CD62L (MEL-14), FITC-conjugated anti-mouse CD27 (LG.3A10), APC-conjugated anti-mouse IgD (11-26c.2a), FITC-conjugated anti-mouse MHCII (M5/114.15.2), PE-conjugated anti-mouse CD80 (16-10A1), PE-cy7-conjugated anti-mouse ICOS (C398.4A), APC-conjugated anti-mouse CX3CR1 (SA011F11), APC-conjugated anti-mouse IFN-γ (XMG1.2), Alexa Fluor 647-conjugated anti-mouse IL-21 (BL25168), PerCP-cy5.5-conjugated anti-mouse GM-CSF (MP1-22E9), PE-conjugated anti-mouse IL-1 (ALF-161), and APC-conjugated anti-mouse IL-6 (MP5-20F3) were purchased from BioLegend (San Diego, CA, USA).

### Histology Studies

Lungs were removed from mice, perfused three times with 0.01 M phosphate-buffered saline (pH = 7.4), fixed in 10% formalin, embedded in paraffin, and sectioned. Sections were then examined by light microscopy after standard hematoxylin-eosin (H&E) staining for visualization of cellular changes under microscopy (Olympus ix71).

### Immunofluorescence Staining

Paraffin sections of lung tissues from wild type (WT) and infected mice were rehydrated and boiled in Sodium citrate buffer (pH 6.0) for 30 min to induce antigen retrieval. After washing, tissue sections were blocked with 10% goat serum, followed by staining with rabbit anti-mouse CD3 antibody (Abcam) and hamster anti-mouse γδT antibody (Santa Cruz Biotechnology) at 4°C overnight. Sections were washed and incubated with Alexa Fluor 555–conjugated anti-rabbit IgG plus Alexa Fluor 488–conjugated anti-hamster IgG (Beyotime, Shanghai, China) for 30 min at 37°C in the dark. After a final washing, cover slips were mounted onto slides with fluoroshield mounting medium with DAPI (Abcam). Images were captured with Olympus microscope BX53 and processed with LSM Image Examiner software (Zeiss).

### Cell Surface Staining

Cells were washed in PBS and blocked in PBS buffer containing 1% BSA for 30 min. Cells were then stained for 30 min at 4°C in the dark with conjugated antibodies specific for the cell surface antigens. Expression phenotypes of antibody-labeled lymphocytes (1 × 10^6^ cells per run) were analyzed using flow cytometry (Beckman CytoFLEX), and the results were analyzed using the CytExpert 1.1 (Beckman Coulter Inc.). The region of single nuclear cells was gated to ensure the dead cell and doublet exclusion. Isotype-matched controls for cell surface markers were included in each staining protocol.

### Intracellular Cytokine Staining

Single lymphocyte suspensions were isolated from the lung, and the cell concentration was adjusted to 1.5 × 10^6^/ml. Cells were then stimulated with phorbol 12-myristate 13-acetate (PMA) (20 ng/ml, Sigma) and ionomycin (1 μg/ml, Sigma) for 5 h (37°C, 5% CO_2_). Brefeldin A (BFA, 10 μg/ml, Sigma) was added during the last 4 h of incubation. Cells were washed twice in PBS and stained for 30 min at 4°C in the dark with conjugated antibodies specific for the cell surface antigens CD3 and γδ TCR. Cells were fixed with Fixation and Permeabilization Solution (BD Biosciences) for 20 min at 4°C in the dark and permeabilized overnight at 4°C in PBS buffer containing 0.1% saponin (Sigma), 1% BSA, and 0.05% NaN_3_. Next, cells were stained with conjugated antibodies that specific for each cytokine. Expression phenotypes of antibody-labeled lymphocytes (1.5 × 10^6^ cells per run) were analyzed using flow cytometry (Beckman CytoFLEX), and the results were analyzed using CytExpert 1.1 (Beckman Coulter Inc.). The region of single nuclear cells was gated to ensure the dead cell and doublet exclusion. Isotype-matched controls for cytokines were included in each staining protocol.

### Cell Culture

Single suspensions were isolated from the lungs of normal and naïve and infected WT and Vδ^−/−^ mice). Cell concentration was adjusted to 2 × 10^6^/ml, and cells were stimulated with anti-CD3 + anti-CD28, soluble egg antigen (SEA) + anti-CD28, and soluble worm antigen (SWA) + anti-CD28; a non-stimulated group was used as a negative control. After mixing well, 200 μl was added to each well in 96-well-flat-bottomed ELISA plates, with each condition having three replicates. Cells were incubated in a cell incubator at 37°C and 5% CO_2_ for 72 h. The supernatant was collected and stored at −20°C.

### ELISA

Levels of IFN-γ and IL-4 in cell supernatants of cultured cells were analyzed by ELISA according to the manufacturer's instructions (555232, BD, 551866, BD). Briefly, the capture antibody was coated on the wells of ELISA plates at 4°C overnight. Wells were washed three times with PBST (0.05% Tween 20 contained PBS) and blocked using 10% fetal calf serum (FCS) in PBS at 37°C for 1 h. Wells were washed three times with PBST again. Supernatants were added and incubated at 37°C for 2 h. After washing, horseradish peroxidase (HRP) linked antibodies were added to wells for 1 h. After further washing, substrates were added and incubated for 20–30 min. Then, wells were read at 450 nm using a microplate reader (Moder ELX-800, BioTek).

### IgG Detection

Sera were extracted from both naïve and infected WT or Vδ^−/−^ mice from the inner canthal vein and cryopreserved at −20°C. SEA and SWA were diluted with sterile PBS solution to 80 μg/ml, and 100 μl of diluted SEA or SWA was added to each well in 96-well ELISA plates at 4°C overnight. The next day, ELISA plates were washed with PBS-T (containing 0.05% Tween) three times. Then, 200 μl of blocking solution (10% calf serum) was added to each well and incubated at 37°C for 2 h. ELISA plates were washed with PBST for three times. 100 μl serum diluted with blocking solution (1:1,000, 1:10,000, and 1:100,000) was added to each well. After 1 h incubation at 37°C, ELISA plates were washed with PBS-T three times, and 100 μl HRP-conjugated anti-mouse IgG detection antibody (1:1,000 dilution) was added for each well and incubated at 37°C for 1 h. ELISA plates were then washed again with PBS-T for five times before adding 100 μl of TMB to each well. After 30 min incubation at 37°C, 50 μl of 10% H_2_SO_4_ was added to each well to terminate the color reaction. Absorbance values of each well were measured by the microplate reader at 450 nm.

### Statistics

Data were analyzed using SPSS 21.0. Statistical evaluation of the difference between means was assessed using one-way ANOVA. *P* < 0.05 was considered to be statistically significant.

## Results

### *S. japonicum* Infection Induces γδ T Cells in the Lung

**Figure 1:**
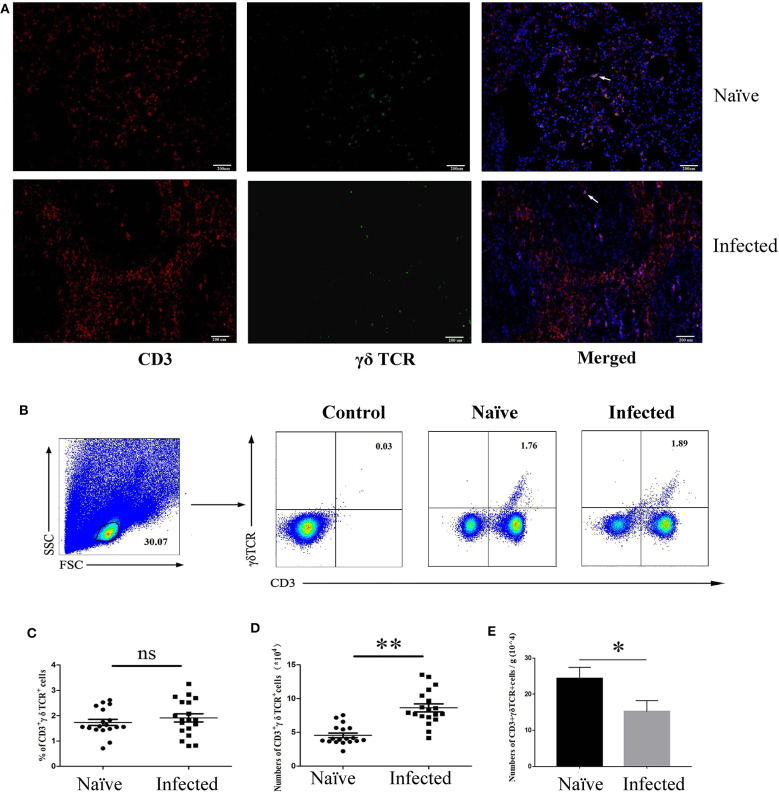
Distribution and content of γδ T cells in *S. japonicum*-infected mouse lungs. Female C57BL/6 mice were infected with 40 ± 5 *S. japonicum cercariae* per mouse, and 5–6 weeks after infection, mice were euthanized. **(A)** Fluorescence staining was performed on paraffin sections of lung tissue from infected mice. CD3 (Red) and γδ TCR (Green) are identified by different fluorescence, and nuclei are visualized using DAPI staining (Blue). A representative experiment from three independent experiments was shown. Scale bar, 200 μm. **(B)** Pulmonary cells were stained with anti-CD3 and anti-γδ TCR mAbs. The percentage of CD3^+^γδTCR^+^ cells in the lungs of naïve and infected mice were analyzed by FCM. One representative experiment was shown. The percentage **(C)** and absolute number **(D)** of CD3^+^γδTCR^+^ cells per mouse, and the number of γδ T cells in per gram of lung tissues **(E)** were calculated from three independent experiments with 5–6 samples, respectively. **P < 0.01, *P > 0.05, ns *P* > 0.05.

To determine the existence of γδ T cells in the lung of *S. japonicum*-infected mice, C57BL/6 mice were euthanized, and the lungs were removed 5–6 weeks after *S. japonicum* infection. Paraffin sections were made and stained with fluorescence-labeled monoclonal antibodies against mouse CD3 and γδ TCR, as well as DAPI, as described in the section Materials and Methods. As shown in Figure 1A, some CD3^+^γδTCR^+^ cells (yellow fluorescence) were present in both naïve and *S. japonicum*-infected mice.

Single cell suspensions were also prepared from the lungs of both naïve and *S. japonicum*-infected mice. Absolute cell numbers were quantified via microscopy, and the percentage of CD3^+^γδTCR^+^ cells was determined by FACS (Figure 1B). As shown in Figure 1C, there was no significant difference in percentages of CD3^+^γδTCR^+^ cells between naïve and infected mice (naïve: 0.92 ± 0.124%, infected: 0.86 ± 0.162%, *P* > 0.05); however, the absolute number of CD3^+^γδTCR^+^ cells after infection was significantly increased (Figure 1D, *P* < 0.01), and the number of γδ T cells in per gram of lung tissue was decreased (Figure 1E, *P* < 0.05).

### Phenotypic Changes in Pulmonary γδ T Cells

**Figure 2:**
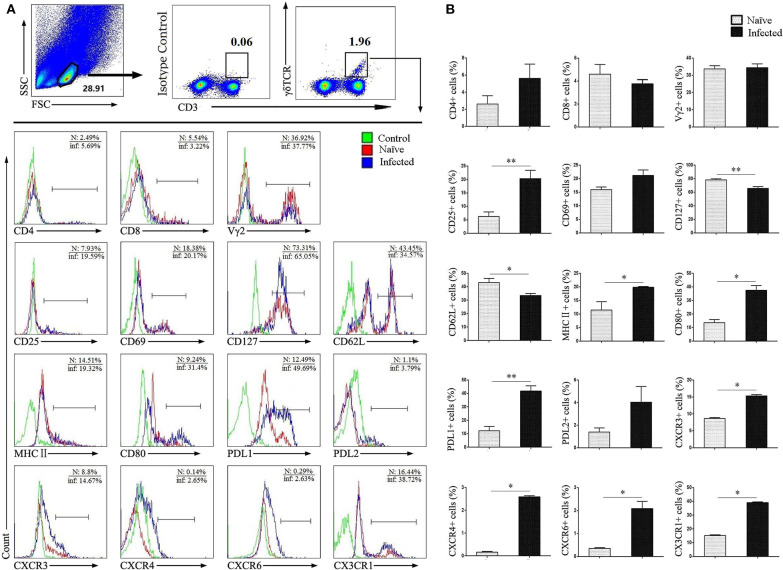
Surface molecule expression on pulmonary γδ T cells. Single lung cell suspensions were separated from naïve and infected mice, and cells were stained with monoclonal antibodies against mice: CD3, γδTCR, CD4, CD8, Vγ2, CD25, CD69, CD127, CD62L, MHC II, CD80, PDL1, PDL2, CXCR3, CXCR4, CXCR6, and CX3CR1. **(A)** Representative FACS analysis of three independent experiments is shown. **(B)** Percentages of surface molecules calculated from FACS analysis, a representative experiment from five independent experiments was shown. The error bars are SD, **P < 0.01, *P < 0.05.

To study the characteristics of CD3^+^γδTCR^+^ cells after infection, single lung cells from both normal naïve and infected mice were stained with different fluorescence labeled factors: CD3, γδ TCR, CD4, CD8, Vγ2, CD25, CD69, CD127, CD62L, MHCII, CD80, PDL1, PDL2, CXCR3, CXCR4, CXCR6, and CX3CR1. Cells were detected by flow cytometry, and the differences were compared. As shown in Figures 2A,B, CD3^+^γδTCR^+^ cells were gated first, and the expression of cell surface molecules was examined. We observed that the expression of CD25, MHCII, CD80, and PDL1 was significantly increased after infection (*P* < 0.05). Differences in the expression of chemokine receptors CXCR3, CXCR4, CXCR6, and CX3CR1 were also significant (*P* < 0.05), but the percentage of positive cells was limited. In contrast, the expression of CD127 and CD62L on γδ T was slightly decreased in infected mice (*P* < 0.05), while there was no significant difference in the expression of CD4, CD8, Vγ2, CD69, and PDL2 (*P* > 0.05).

### Cytokines Released by Pulmonary γδ T Cells

**Figure 3:**
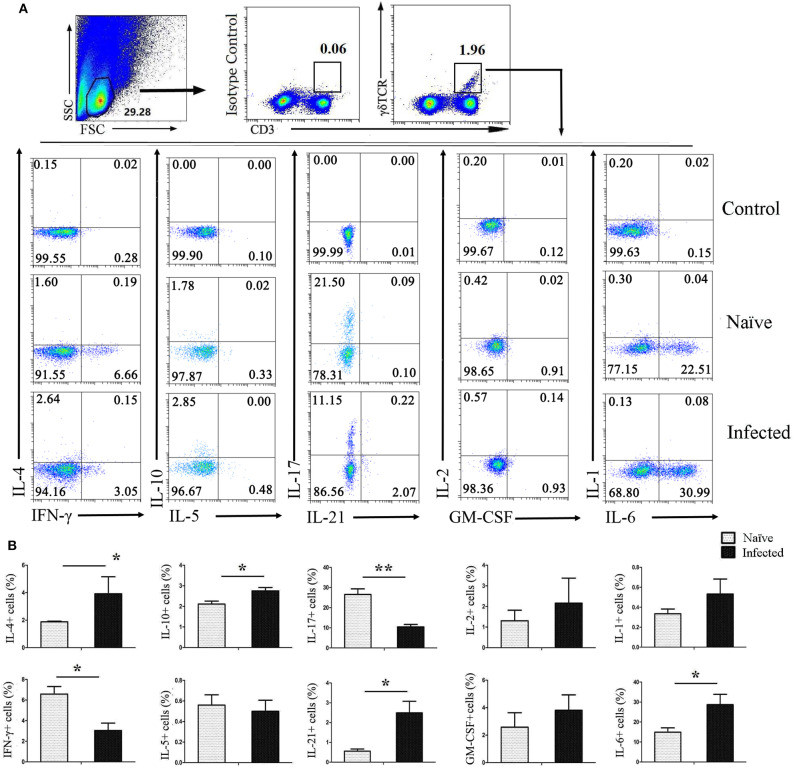
Cytokines released by pulmonary γδ T cells. C57BL/6 mice were infected with 40 ± 5 *S. japonicum cercariae* per mouse, and 5–6 weeks after the infection, mice were euthanized. Single cell suspensions of lung cells were stimulated with PMA and ionomycin. Cytokine (IL-4, IFN-γ, IL-10, IL-5, IL-17, IL-21, IL-2, GM-CSF, IL-1, and IL-6) expression was detected in CD3^+^γδTCR^+^ cells by FACS analysis. **(A)** A representative of four independent experiments with 5–6 mice per group is shown. **(B)** Average expression of different cytokines on CD3^+^γδTCR^+^ cells was calculated by FACS data. The error bars are SD, **P < 0.01, *P < 0.05.

To investigate the ability of CD3^+^γδTCR^+^ cells to secrete cytokines after infection, pulmonary single cell suspensions were isolated from both naïve and infected mice and stimulated with PMA and ionomycin for 5 h, followed by staining for intracellular cytokines. As shown in Figure 3A, CD3^+^γδTCR^+^ cells were gated first, and then the expression of IL-4, IFN-γ, IL-10, IL-5, IL-17, IL-21, IL-2, GM-CSF, IL-1, and IL-6 were detected. As shown in Figure 3B, the expression of IL-4, IL-10, IL-21, and IL-6 on CD3^+^γδTCR^+^ cells in naïve mouse lung was significantly lower than in infected mouse lung (*P* < 0.05). However, the percentage of IFN-γ-expressing CD3^+^γδTCR^+^ cells in naïve mice was higher than in infected mice (*P* < 0.05), and the percentage of IL-17-expressing CD3^+^γδTCR^+^ cells in naïve mice was higher than in infected mice (*P* < 0.01). There was no significant difference in the expression of IL-5, IL-2, GM-CSF, or IL-1 between naïve and *S. japonicum*-infected lung-resident CD3^+^γδTCR^+^ cells (*P* > 0.05).

### Pathological Changes of the Lung in Vδ^-/-^ Mice

**Figure 4:**
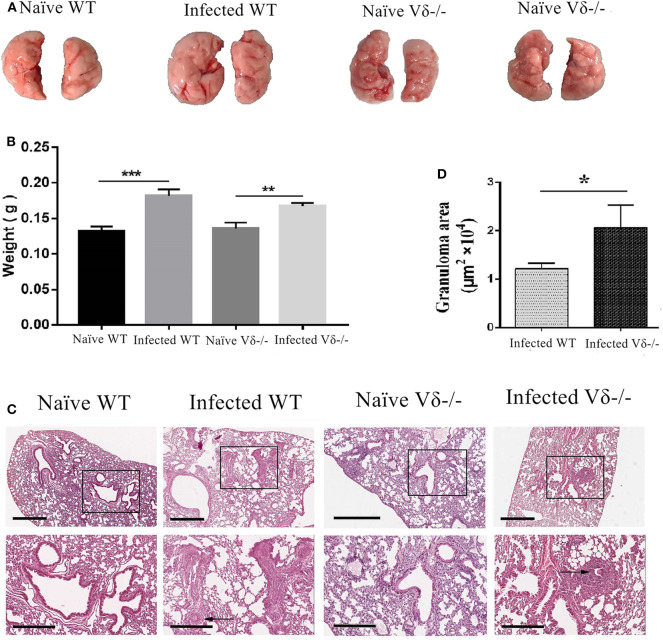
Pathological changes in the lung of infected Vδ^−/−^ mice. C57BL/6 and Vδ^−/−^ mice were infected with 40 ± 5 *S. japonicum cercariae* per mouse. Five to six weeks after infection, mice were euthanized. **(A)** Lung appearance in naïve and infected WT and Vδ^−/−^ mice was compared. **(B)** The weight of the lung from naïve and infected WT and Vδ^−/−^ mice was compared. **(C)** Hematoxylin and eosin staining were performed on paraffin sections of lung tissue. Scale bar, 600 μm (upper), 300 μm (lower). **(D)** The area of granuloma in infected WT and Vδ^−/−^ mice was compared. A representative of three independent experiments with 5–6 mice per group is shown. The error bars are SD, *P < 0.05, **P < 0.01, ***P < 0.001.

To elucidate the role of γδ T cells in *S. japonicum* infection, both WT and Vδ^−/−^ mice were infected at the same time. Mice were euthanized 5–6 weeks after infection, and lungs were removed. As shown in Figure 4A, compared with infected WT mice, the size of the lungs in infected Vδ^−/−^ mice was similar. However, the color was paler, and there was no visible granuloma in the lungs of naïve mice. Although the weight of the lung from infected mice were higher than that from the uninfected mice (*P* < 0.05), there was no obvious different in the weight of lung between the infected wild type (WT) and infected Vδ^−/−^ mice (Figure 4B). As shown in Figure 4C, lung biopsy (HE staining) was observed, and the structure of lung tissue in naïve mice was clear with a uniform distribution of lung cells, but inflammatory cell aggregation was found in the lung tissue of infected and knockout mice. Moreover, the area of granuloma was calculated as described in the section Materials and Methods. As shown in Figure 4D, the area of granuloma in lung tissue was significantly increased in the infected Vδ^−/−^ mice (*P* < 0.05).

### Effect of γδ T Cells on T Cells

**Figure 5:**
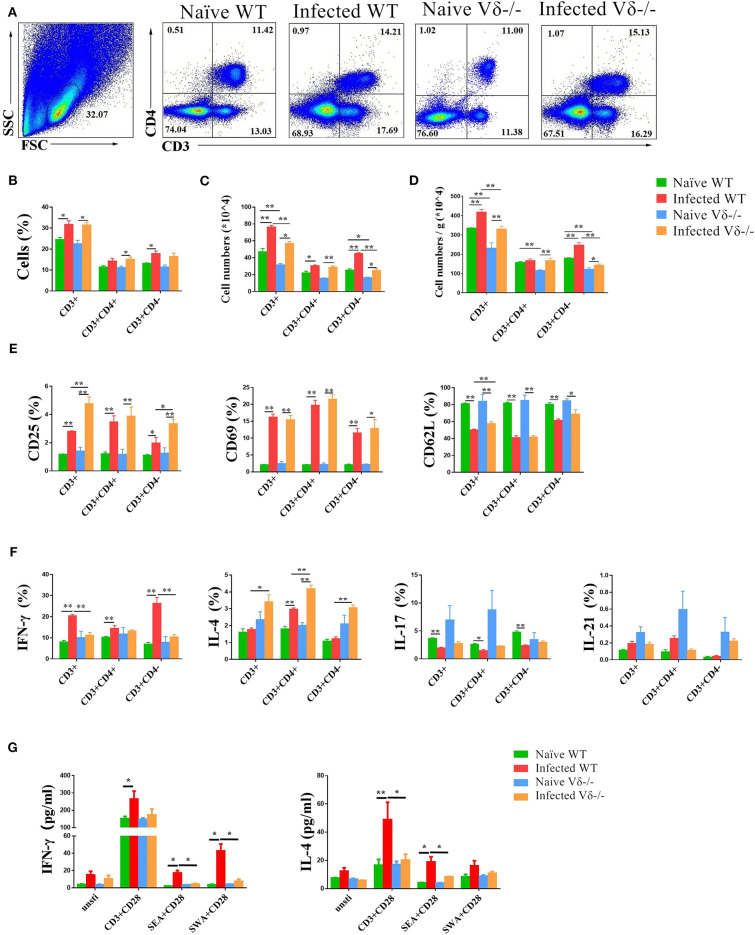
The effect of γδ T cells on T cells. C57BL/6 and Vδ^−/−^ mice were infected with 40 ± 5 *S. japonicum cercariae* per mouse or not, and mice were euthanized after 5–6 weeks of infection. Single lung cell suspensions were separated. **(A–D)** Percentages and absolute numbers of CD3^+^ T cells, CD3^+^CD4^+^ and CD3^+^CD4^−^ cell population were counted. **(E)** Activation of CD3^+^ T cells, CD3^+^CD4^+^ T cells and CD3^+^CD4^−^ cells in naïve and infected WT and Vδ^−/−^ mice. **(F)** Expression of IL-4, IFN-γ, IL-17, and IL-21 in CD3^+^ T cells, CD3^+^CD4^+^ T cells and CD3^+^CD4^−^ cells in each groups of mice. **(G)** Secretion of IL-4, IFN-γ, and IL-17 in the supernatant of lung cells cultured under different stimulants. Single lung cell suspensions of each group of mice were prepared and cultured *in vitro* with anti-CD3 + anti-CD28, SEA + anti-CD28, and SWA + anti-CD28, respectively. The supernatants were collected after 72 h. The secretion of IFN-γ and IL-4 was detected by ELISA. Data represent the average of three independent experiments with 5–6 mice per group. The error bars are SD, **P < 0.01, *P < 0.05.

To investigate the modulating role of γδ T cells on T cells in the lung of naïve, infected WT and Vδ^−/−^ mice, the content, active molecular expression and cytokine producing ability of T cells were compared (Figure 5). The results indicated that the percentage of CD3^+^ cells in infected (both WT and Vδ^−/−^) mice were higher than the uninfected mice (*P* < 0.05, Figure 5A). The percentage of CD3^+^CD4^−^ in infected WT mice, and CD3^+^CD4^+^ cells in infected Vδ^−/−^ mice were higher than that in the uninfected control mice, too (*P* < 0.05, Figure 5B). The absolute number of CD3^+^, CD3^+^CD4^+^, and CD3^+^CD4^−^ cells in infected mice was higher than in uninfected mice (*P* < 0.05, Figure 5C). However, the absolute number of CD3^+^ and CD3^+^CD4^−^ cells in infected Vδ^−/−^ mice were lower than in infected WT mice (*P* < 0.05, Figure 5C). Additionally, the number of T cells in per gram lung tissue were calculated and compared. As shown in Figure 5D, the increasing number of T cells of per gram lung tissue could be found in the infected groups. The changes of the numbers of T cells, and radio of CD3^+^, CD3^+^CD4^+^, and CD3^+^CD4^−^ cells in the infected Vδ^−/−^ mice were lower than that in infected WT mice (*P* < 0.05).

To examine the role of γδ T cells on activation of T cells, the expression of CD25, CD69, and CD62L on CD3^+^, CD3^+^CD4^+^, and CD3^+^CD4^−^ cells in both naïve and infected WT and Vδ^−/−^ mice were measured by FACS (Supplementary Figures 1A,B). As shown in Figure 5E, the expression of CD25 and CD69 on CD3^+^, CD3^+^CD4^+^, and CD3^+^CD4^−^ cells was significantly increased in both infected WT and infected Vδ^−/−^ mice compared to the naïve control mice (*P* < 0.05), and the expression of CD25 on CD3^+^ and CD3^+^CD4^−^ cells was significantly increased in infected Vδ^−/−^ mice compared to the infected WT mice (*P* < 0.05). The expression of CD62L on CD3^+^, CD3^+^CD4^+^, and CD3^+^CD4^−^ cells were significantly decreased in the infected mice (*P* < 0.05). The expression of CD62L on CD3^+^ cells was significantly decreased in infected WT mice compared to infected Vδ^−/−^ mice (*P* < 0.05).

To examine the role of γδ T cells on the function of T cells, single cell suspensions from naïve and infected WT and Vδ^−/−^ mice were prepared, stimulated, and stained as described in the section Materials and Methods. IL-4, IFN-γ, IL-17, and IL-21 were detected on CD3^+^, CD3^+^CD4^+^ and CD3^+^CD4^−^ cells (Supplementary Figures 1C–E). As shown in Figure 5F, the expression of IL-4 in the cell from infected mouse were higher than the naïve mice, especially in CD3^+^CD4^+^ cells (*P* < 0.05), and the expression of IL-4 in CD3^+^, CD3^+^CD4^+^, and CD3^+^CD4^−^ cells from the infected Vδ^−/−^ mice was higher than that in infected WT mice (*P* < 0.05). The expression of IFN-γ in the cell from infected mouse were also higher than the uninfected mice, especially in CD3^+^CD4^−^ cells (*P* < 0.05), and the percentage of IFN-γ expressing CD3^+^ and CD3^+^CD4^−^ cells from infected Vδ^−/−^ mice was lower than in infected WT mice (*P* < 0.05). There was no difference in the expression of IL-17 and IL-21 on CD3^+^, CD3^+^CD4^+^ or CD3^+^CD4^−^ cells between infected WT and infected Vδ^−/−^ mice (*P* > 0.05).

To further explore the effects of γδ T cells on the production of cytokines in other lung cells, single lung cell suspensions were isolated from naïve and infected WT and Vδ^−/−^ mice. Cells were cultured *in vitro* for 72 h with different stimulators (anti-CD3 plus anti-CD28, SEA plus anti-CD28, or SWA plus anti-CD28) and compared to non-stimulated negative controls. Levels of IL-4, IFN-γ, and IL-17 in the culture supernatants were detected by ELISA. As shown in Figure 5G, in the anti-CD3 + anti-CD28 and SEA + anti-CD28 stimulated group, levels of IL-4 secreted by lung cells in infected WT mice were significantly higher than in naïve WT mice (*P* < 0.05) and the infected Vδ^−/−^ mice (*P* < 0.05). In response to the three stimulation conditions, levels of IFN-γ secreted by lung cells in infected WT mice were significantly higher than in naïve WT mice (*P* < 0.05). And in the SEA + anti-CD28 and SWA + anti-CD28 stimulated groups, levels of IFN-γ secreted by lung cells in infected WT mice were higher than in infected Vδ^−/−^ mice (*P* < 0.05).

### Effect of γδ T Cells on B Cells

**Figure 6:**
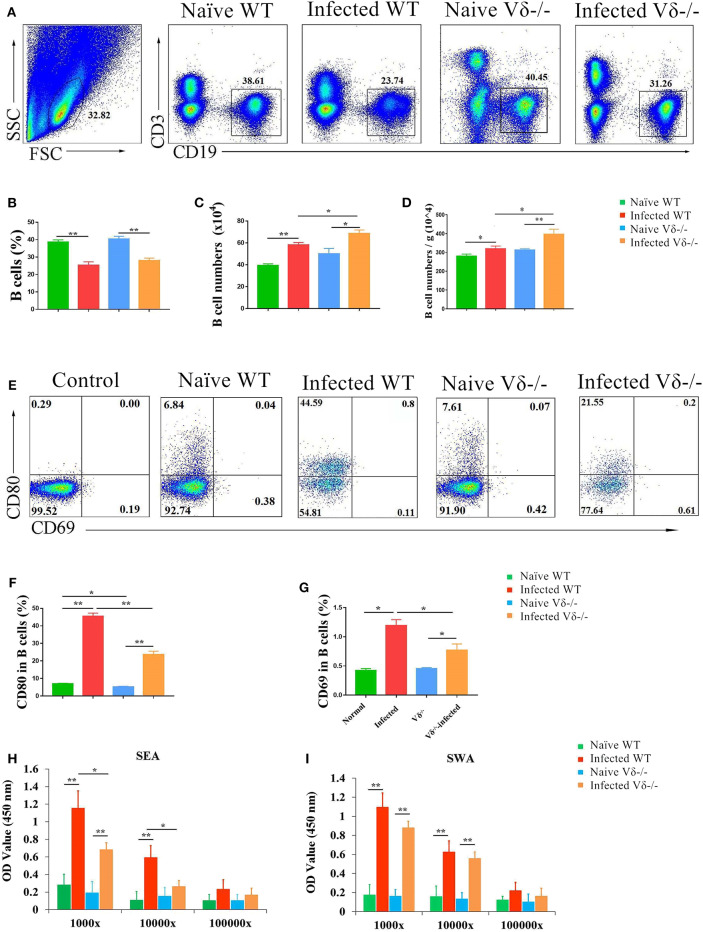
The effect of γδ T cells on B cells. C57BL/6 mice and Vδ^−/−^ mice were infected with 40 ± 5 *S. japonicum cercariae* per mouse. Five to six weeks after infection, mice were euthanized. Single lung cell suspensions were separated. **(A–D)** Percentage and number of B cells in the lung of naïve and infected WT and Vδ^−/−^ mice were detected. **(E–G)** Expression of CD69 and CD80 on B cells in naïve and infected WT and Vδ^−/−^ mice. Blood were collected, and serum was diluted in different proportion (1,000, 10,000, and 100,000 folds). The levels of IgG in the serum of four groups were detected by ELISA. **(H)** Soluble egg antigen (SEA)-specific IgG is shown. **(I)** Soluble worm antigen (SWA)-specific IgG is shown. Data represent the average of three independent experiments with 5–6 mice per group. The error bars are SD, **P < 0.01, *P < 0.05.

To investigate the effect of γδ T cells on B cells in the lung of *S. japonicum*-infected mice, single nuclear cell solutions from both naïve and infected WT and Vδ^−/−^ mice were prepared as described in the section Materials and Methods, and the content of CD19^+^ B cells was compared by FACS (Figure 6A). Although the percentage of CD19^+^ B cells in infected (both WT and Vδ^−/−^) mice was lower than in naïve mice (*P* < 0.05, Figure 6B), the absolute number of CD19^+^ B cells was significantly increased in the infected (both WT and Vδ^−/−^) mice (*P* < 0.05, Figure 6C). The absolute number of CD19^+^ B cells in the infected Vδ^−/−^ mice was higher than in infected WT mice (*P* < 0.05), and there were no significant differences in the percentage and number of B cells between naïve C57BL/6 and naïve Vδ^−/−^ mice (*P* > 0.05). Additionally, the number of B cells in per gram lung tissue were calculated and compared. As shown in Figure 6D, obvious increasing in the number of B cells (per gram) could be found in the infected groups (*P* < 0.05), and the ratio in the infected Vδ^−/−^ mice was higher than that in infected WT mice (*P* < 0.05).

Next, the expression levels of CD80, and CD69 were examined on B cells from each group of mice B by FACS (Figure 6E). As shown in Figures 6F,G, the expressions of CD69 and CD80 on B cells in infected (both WT and Vδ^−/−^) mice were significantly higher than in uninfected mice (*P* < 0.05), and the expression of CD69 and CD80 on B cells from infected Vδ^−/−^ mice was lower than from infected C57BL/6 mice (*P* < 0.05). In addition, levels of SEA and SWA specific IgG in serum from different experimental groups were detected by ELISA. As shown in Figure 6H, levels of SEA-specific IgG in Vδ^−/−^ mice were significantly decreased compared to infected WT mice (*P* < 0.05). However, there were no differences in the levels of SWA-specific IgG between infected and Vδ^−/−^ mice (*P* > 0.05, Figure 6I).

## Discussion

γδ T cells is a kind of innate immune cells apply more to γδ T cells from the intestine (24). In this study, the role of γδ T cells in the lungs of C57BL/6 mice infected with the *S. japonicum* was evaluated. As shown in Figure 1, the absolute number of γδ T cells was significantly increased, though the percentage did not change (Figure 1), indicating that γδ T cells accumulate in the lung and may play roles in the progress of *S. japonicum* infection.

Next, the phenotype of γδ T cells was examined, and differences between naïve and infected mice were compared. As shown in Figure 2, no difference was detected in the expression of γδ T cell subset-associated molecules CD4, CD8, and Vγ2 as reported (25, 26) (*P* > 0.05). CD25, CD69, CD127, and CD62L are T cell activation-associated molecules that mediate the proliferation and migration of leukocytes at inflammatory sites and the recirculation of lymphocytes between blood and lymphoid tissues (27). Our results demonstrated that CD25 is increased (*P* < 0.01) and CD62L is decreased (*P* < 0.05) on the surface of infected γδ T cells (Figure 2), which indicated that these pulmonary γδ T cells were activated. These data further confirm that γδ T cells are involved in pulmonary inflammation in infected mice.

MHC II and co-stimulatory molecules CD80 are primarily expressed on the surface of antigen presenting cells (APCs), which could facilitate activation of antigen-specific T cells (28). It was reported that γδ T cells could become APCs in some conditions (9). Our results showed that MHC II and CD80 expression on γδ T cells was significantly increased in response to infection (*P* < 0.05), suggesting that the γδ T cells might be presented as APCs, which mediate the immune response in the lung of infected mice. PDL1 and PDL2 are inhibitory receptors on APCs that bind to PD-1 molecules expressed on active T cells, inducing T cell functional exhaustion (29). We found that expression of PDL1 was significantly increased on γδ T cells from infected mice (*P* < 0.05, Figure 2). It further suggested that γδ T cells might act as a type of APC which mediating the immune response in the lung of infected mice (30). In addition, chemokines and their specific receptors play important roles in immune response mediated lymphocyte accumulation. It was reported that chemokine receptors CXCR3 and CXCR4 related to lung function damage in patients with systemic sclerosis (31). In allergic asthma, CX3CR1 expression regulates Th2 and Th1 cell survival in the inflammatory lung, while, in atopic dermatitis, it regulate Th2 and Th1 cell retention into the inflammatory site (32). In this study, the expression of CXCR3, CXCR4, CXCR6, and CX3CR1 on γδ T cells was found up-regulated, especially CX3CR1 (*P* < 0.05, Figure 2). It suggested that the elevated expression of chemokine receptors might account for the increased number of γδ T cells in the lung, and CX3CR1 might be one of the factors influencing the function of γδ T cells in the lung of *S. japonicum-*infected mice.

As an important innate lymphocyte, γδ T cells secrete a variety of cytokines to regulate the immune response (11). To further explore the roles of γδ T cells in the lung of *S. japonicum-*infected mice, the profile of inflammatory cytokines was examined and compared between naïve and infected mice. The results indicated that γδ T cells secrete more Th2 cytokines (IL-4, IL-10) and fewer Th1 cytokines (IFN-γ) in response to infection (Figure 3). It suggested that the type 2 immune response is facilitated by increased type 2 cytokine expression from γδ T cells. IL-17 is a pro-inflammatory cytokine that activates neutrophils, macrophages, and cytotoxic lymphocytes to enhance antimicrobial immunity (33, 34). γδ T cells are reportedly the primary source of IL-17 in early infection rather than CD4^+^ T cells (35). However, the percentage of IL-17^+^ γδ T cells decreased significantly after infection (*P* < 0.05). This is in contrast to the results we observed in the liver of *S. japonicum-*infected mice (36). This might relate to the different immune microenvironments between liver and lung. IL-21 is a multi-effect cytokine that promotes the activation and differentiation of B cells (37). In this study, the results showed that γδ T cells from infected mice expressed higher IL-21 (*P* < 0.05), indicating that γδ T cells could modulate the response of B cells through this pathway.

To validate the role of γδ T cells in *S. japonicum* infection-induced pulmonary damage, Vδ gene knockout (Vδ^−/−^) mice were infected with *S. japonicum*. As shown in Figure 4, although no obvious morphological differences were detected in lungs, the area of granuloma in the lung tissue of Vδ^−/−^ mice was significantly increased (*P* < 0.05), validating the idea that γδ T cells might inhibit the accumulation of inflammatory cells in the lung of *S. japonicum-*infected mice.

Furthermore, T cell response was compared between *S. japonicum-*infected WT and Vδ^−/−^ mice. As shown in Figure 5, decreased CD3^+^CD4^−^ cells numbers (*P* < 0.05), percentage of IFN-γ-secreting CD3^+^CD4^−^ cells (*P* < 0.05), and increased percentage of IL-4-secreting CD3^+^CD4^+^ cells (*P* < 0.05) could be found in infected Vδ^−/−^ mice, compared to infected WT mice. It suggests that γδ T cells could enhance the differentiation of Th1 cell in this model. This result is in agreement with previous experiments, which demonstrated that γδ T cells have a protective effect in mouse infection and injury models (20, 38). Consistent to the FACS result, the results of *in vitro* cell culture experiments indicated that levels of IFN-γ decreased in Vδ^−/−^ mice in response to both SEA and SWA, suggesting that γδ T cells could enhance type 1 immune response in infected mice (Figure 5G). Although the percentage of IL-4-secreting CD3^+^CD4^+^cells was increased in infected Vδ^−/−^ mice as showed in Figure 5F, the levels of IL-4 induced by CD3 plus CD28, and SEA plus CD28 treatment were significantly decreased in infected Vδ^−/−^ mice (*P* < 0.05). It implies that γδ T cells could enhance the type 2 immune response in infected mice, which might related to the molecules and cytokines expressing on the infection induced γδ T cells as showed in Figures 2, 3.

Additionally, B cell response was investigated in infected Vδ^−/−^ mice as shown in Figure 6. The absolute numbers of B cells per gram of lung tissue of B cell in the infected Vδ^−/−^ mice were increased compared to the infected WT mice (*P* < 0.05, Figures 6C,D). It suggests that γδ T cells can modulate the proliferation of B cells. CD69 and CD80 are B cell activation and functionally related molecules (39, 40). The expression of these molecules was detected on B cells, and the results (Figures 6E–G) revealed that only expression of CD80 and CD69 on B cells was decreased in the lung of infected Vδ^−/−^ mouse compared to infected WT mice (*P* < 0.05). These results suggest that γδ T cells can enhance B cell activation. In addition, levels of SEA-specific IgG in the serum of Vδ^−/−^ mice decreased significantly (Figure 6H, *P* < 0.05), whereas SWA-specific IgG levels remained unchanged (*P* > 0.05, Figure 6I). This is consistent with the above results and indicates that γδ T cells primarily mediate SEA-induced B cell response in the course of *S. japonicum* infection. Similarly, γδ T cells was found to enhance antibody production and recovery from *semliki forest virus* (SFV) demyelinating disease (41).

In conclusion, this study investigated the phenotypic and functional characteristics of γδ T cells in the lung of *S. japonicum-*infected mice, and we found that γδ T cells significantly adjust the type 2 immune response during the course of *S. japonicum* infection by the expression of specific molecules and production of specific cytokines.

## Data Availability Statement

The raw data supporting the conclusions of this article will be made available by the authors, without undue reservation, to any qualified researcher.

## Ethics Statement

The animal study was reviewed and approved by the institutional animal care and use committee of Guangzhou Medical University.

## Author Contributions

HC, HX, and CJ performed most experiments and analyzed data with the support from JH. CJ, YF, QW, and QY performed animal experiment. AX and SX performed parasite infection experiment. HQ and YQ contributed to scientific planning. ZY and JH oversaw and designed the study. JH and JM wrote the manuscript.

## Conflict of Interest

The authors declare that the research was conducted in the absence of any commercial or financial relationships that could be construed as a potential conflict of interest.
