# Supplementary material for: Adjustments of γδ T Cells in the Lung of Schistosoma japonicum-Infected C56BL/6 Mice
Source: Front Immunol. 2020 Jun 4;11:1045. doi: 10.3389/fimmu.2020.01045 (PMC7287124; doi:10.3389/fimmu.2020.01045)
Supplement: Supplementary file 1 [file Data_Sheet_1.doc]

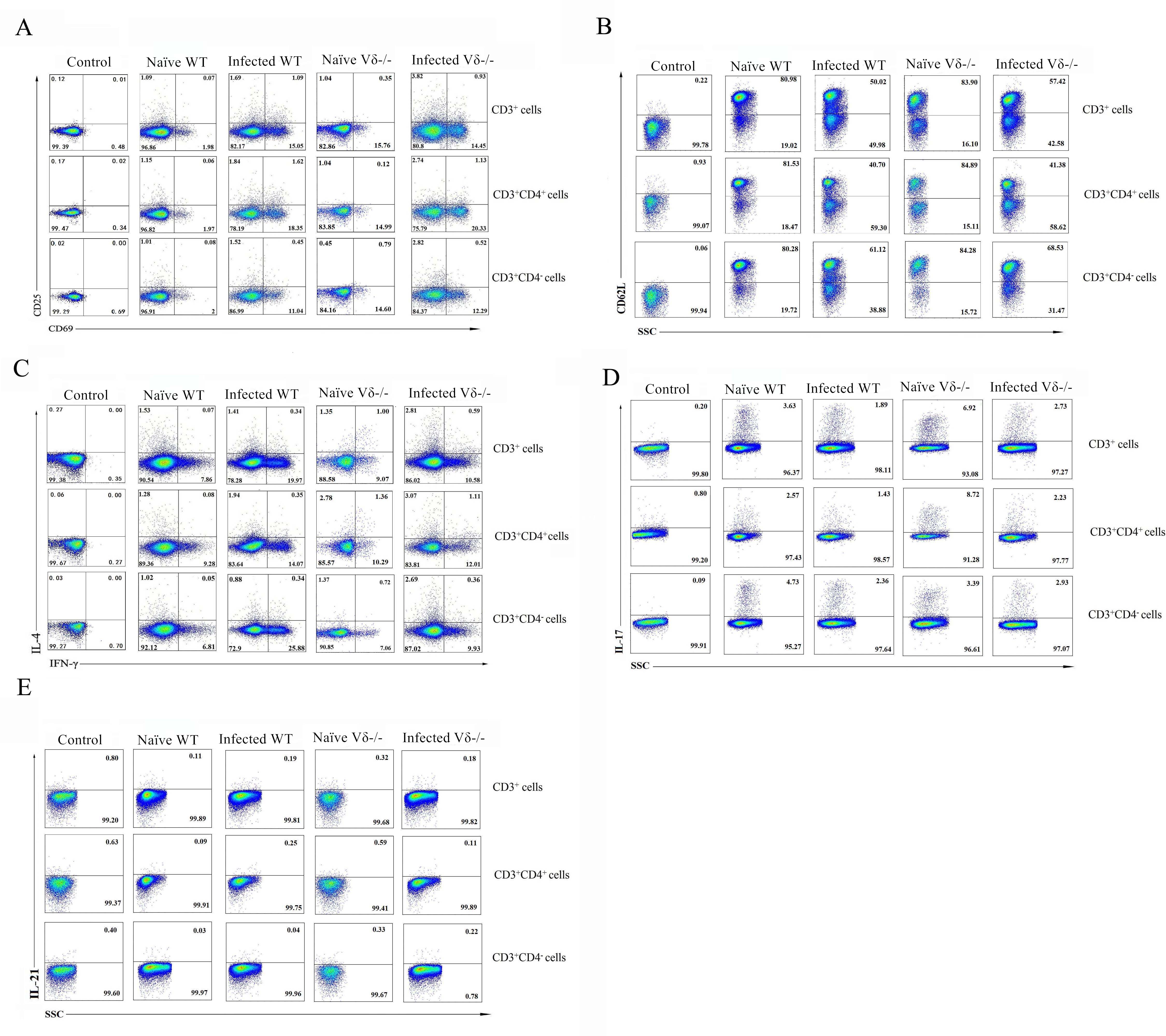


Supplementary Figure 1 The effect of γδ T cells on T cells. C57BL/6 mice and Vδ^-/-^ mice were infected with 40±5 *S. japonicum cercariae* per mouse, and 5-6 weeks after infection, mice were euthanized. Single lung cell suspensions were separated. (A, B) The expression CD25, CD69, and CD62L on CD3^+^ T cells, CD3^+^CD4^+^ T cells and CD3^+^CD4^-^ cells in normal and infected wild type or Vδ^-/-^ mice. (C-E) Expression of IL-4, IFN-γ, IL-17 and IL-21 in CD3^+^ T cells, CD3^+^CD4^+^ T cells and CD3^+^CD4^-^ cells in the four groups of mice. A representation result was shown.
